# Study protocol of “Worth the Walk”: a randomized controlled trial of a stroke risk reduction walking intervention among racial/ethnic minority older adults with hypertension in community senior centers

**DOI:** 10.1186/s12883-015-0346-9

**Published:** 2015-06-15

**Authors:** Ivy Kwon, Sarah Choi, Brian Mittman, Nazleen Bharmal, Honghu Liu, Barbara Vickrey, Sarah Song, Daniel Araiza, Heather McCreath, Teresa Seeman, Sang-Mi Oh, Laura Trejo, Catherine Sarkisian

**Affiliations:** Department of Medicine, Division of Geriatrics, David Geffen School of Medicine at UCLA, 10945 Le Conte Ave, Suite 2339, Los Angeles, CA 90095 USA; Nursing Science, College of Health Sciences, UC Irvine, 100B Berk Hall, University of California Irvine, Irvine, CA 92697 USA; VA Greater Los Angeles Healthcare System, Center for Implementation Practice and Research Support, 16111 Plummer Street, North Hills, CA 91343 USA; UCLA Division of General Internal Medicine and Health Services Research, 911 Broxton Avenue, Los Angeles, CA 90095 USA; Department of Biostatistics at UCLA, CHS 63-037A, 10833 Le Conte Ave, Los Angeles, CA 90095 USA; Department of Neurology at UCLA, 710 Westwood Plaza, C109 RNRC, Los Angeles, CA 90095 USA; Vascular Neurology at Rush University, Professional Office Building, 1725 W. Harrison St, Suite 1121, Chicago, IL 60612 USA; Geriatrics Research Center, Division of Geriatrics, David Geffen School of Medicine at UCLA, 10880 Wilshire Boulevard, Suite 860, Los Angeles, CA 90024 USA; American Heart Association/American Stroke Association, One South Almaden Boulevard, Suite 500, San Jose, CA 95113 USA; City of Los Angeles Department of Aging, 221 N. Figueroa St., Suite 180, Los Angeles, CA 90012 USA; VA Greater Los Angeles Healthcare System Geriatric Research Education Clinical Center (GRECC), Building 220, Room 315 (11G), 11301 Wilshire Blvd, Los Angeles, CA 90073 USA

**Keywords:** Seniors, Ethnic minority, Stroke, Primary prevention, Behavioral intervention, Clinical trial

## Abstract

**Background:**

Stroke disproportionately kills and disables ethnic minority seniors. Up to 30 % of ischemic strokes in the U.S. can be attributed to physical inactivity, yet most Americans, especially older racial/ethnic minorities, fail to participate in regular physical activity. We are conducting a randomized controlled trial (RCT) to test a culturally-tailored community-based walking intervention designed to reduce stroke risk by increasing physical activity among African American, Latino, Chinese, and Korean seniors with hypertension. We hypothesize that the intervention will yield meaningful changes in seniors’ walking levels and stroke risk with feasibility to sustain and scale up across the aging services network.

**Methods/Design:**

In this randomized single-blind wait-list control study, high-risk ethnic minority seniors are enrolled at senior centers, complete baseline data collection, and are randomly assigned to receive the intervention “Worth the Walk” immediately (*N* = 120, intervention group) or in 90 days upon completion of follow-up data collection (*N* = 120, control group). Trained case managers employed by the senior centers implement hour-long intervention sessions twice weekly for four consecutive weeks to the intervention group. Research staff blinded to participants’ group assignment collect outcome data from both intervention and wait-list control participants 1 and 3-months after baseline data collection. Primary outcome measures are mean steps/day over 7 days, stroke knowledge, and self-efficacy for reducing stroke risk. Secondary and exploratory outcome measures include selected biological markers of health, healthcare seeking, and health-related quality of life. Outcomes will be compared between the two groups using standard analytic methods for randomized trials. We will conduct a formal process evaluation to assess barriers and facilitators to successful integration of Worth the Walk into the aging services network and to calculate estimated costs to sustain and scale up the intervention. Data collection is scheduled to be completed in December 2016.

**Discussion:**

If this RCT demonstrates superior improvements in physical activity and stroke knowledge in the intervention group compared to the control group and is found to be sustainable and scalable, Worth the Walk could serve as a primary stroke prevention model for racial/ethnic communities across the nation.

**Trial registration:**

ClinicalTrials.gov NCT02181062; registered on June 30, 2014.

**Electronic supplementary material:**

The online version of this article (doi:10.1186/s12883-015-0346-9) contains supplementary material, which is available to authorized users.

## Background

It is estimated that someone in the U.S. has a stroke every 40 sec and dies of one every 4 min [1]. Racial/ethnic minorities are disproportionately affected by stroke [2, 3], with hypertensive older adults at particularly high risk [1, 4]. Stroke incidence among African Americans and Mexican Americans is higher than among non-Latino whites [1, 5]. African Americans, Latinos and Chinese Americans have higher incidence of hemorrhagic stroke than non-Latino whites [6]. In the U.S., the relative risk of stroke mortality is up to 1.4 higher for Asians compared to non-Latino whites [7, 8]. African Americans [9], Latinos [10, 11], Chinese Americans [12] and Koreans [13] consistently report lower levels of knowledge about stroke and its risk factors. This knowledge gap is a likely contributing factor in observed stroke disparities and worse stroke outcomes for ethnic minorities.

Physical inactivity is a powerful modifiable risk factor for stroke and accounts for up to 30 % of population-attributable ischemic stroke risk in the U.S. [14, 15]. Regular physical activity has been associated with substantially lower stroke risk [16]. Despite the empiric data supporting the benefits of exercise for stroke prevention and other health outcomes including mortality, over 30 % of Americans are physically inactive, with older adults and ethnic minorities being the least active groups [1, 17]. This suggests that there is a tremendous opportunity to reduce population stroke risk and decrease stroke disparities by increasing physical activity among ethnic minority seniors.

To this end, our UCLA scientists are working in close collaboration with the City of Los Angeles (L.A.) Department of Aging (DoA), a local Area Agency on Aging (AAA) overseen by the U.S. Administration for Community Living (ACL), to design, implement and test an effective sustainable program to increase physical activity and decrease stroke risk. Federal congressionally mandated Title III funds are distributed each year to AAAs that use this money to contract with local service providers (including a vast network of senior centers) to provide services to 3 million seniors annually [18]. These services include but are not limited to assistance with meals, transportation, housing, safety and, increasingly, health promotion. Under the leadership of Dr. Kathy Greenlee, the U.S. ACL has advocated for the local AAAs to implement evidence-based health promotion programs such as the Chronic Disease Self- Management Program developed at Stanford University [19]; under the leadership of our collaborator General Manager Laura Trejo, the City of L.A. DoA has been a national leader in this effort to implement evidence-based programs.

Our team is currently testing the effectiveness of a culturally-tailored walking intervention called “Worth the Walk” (WTW) developed for African American, Latino, Chinese and Korean seniors on reducing physical inactivity. Our primary specific aim is: 1) to measure the effectiveness of the intervention in increasing walking levels (mean steps/day measured by pedometers) at the end of the 4 week intervention, and persistence after two months. We hypothesize that WTW will increase physical activity by increasing knowledge about stroke risk factors and improving self-efficacy for reducing stroke risk and being physically active. In addition to the primary study aim, the study has two sub-aims: 2) to explore the relationship between the intervention and biological markers of health including blood pressure, body-mass index, non-HDL cholesterol, glycosylated hemoglobin (HgA1c), and c-reactive protein (CRP); and 3) to examine the relationship between the intervention, self-efficacy, and healthcare seeking behaviors in order to best control stroke risk factors.

Worth the Walk was designed to be sustainable under routine (non-study) conditions, as it utilizes case managers who are already funded through the AAAs. Upon completion of this research study, we will have created a complete set of materials (training manuals, curricula, and accompanying materials) that AAAs across the nation can use to integrate the intervention into their own aging services networks. We will collect critical data on the delivery of WTW and its potential for sustainment and spread beyond the effectiveness trial period and participating sites, with the goal of facilitating its implementation into routine practice throughout the U.S.

## Methods/Design

**Fig. 1:**
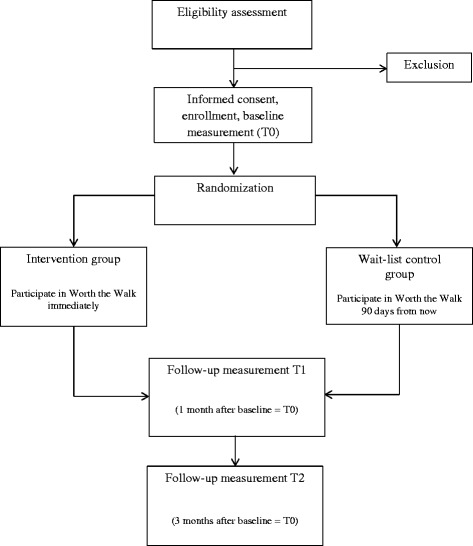
Study design

This study is a single-blind randomized wait-list controlled trial. Participants are randomized within 4 ethnic-specific clusters at senior centers in Los Angeles to either immediate intervention or 3-month wait list (see Fig. 1). The intervention itself lasts 4 weeks. Measures are taken at baseline prior to randomization (T0), 1-month (T1—for the intervention arm, this is immediately following the 4-week intervention), and 3-months (T2—for the intervention arm, this is 2 months after completion of the intervention). Measures for control group participants are taken at the same time points (1 and 3-months after baseline) while they are still on the wait list; after 3-month data collection, all control arm participants are invited to participate in WTW but do not repeat outcome measures.

### Community-based participatory research

This project has been designed and is conducted based on the principles of community-based participatory research, in which academic and community partners are equal members of the study team and participate together in shared decision making on the design, implementation, evaluation and dissemination of research findings [20]. The Los Angeles Community Academic Partnership for Research in Aging (L.A. CAPRA) serves as the infrastructure for this bidirectional community-academic partnered research project. L.A. CAPRA is a collaboration between the University of California Los Angeles Division of Geriatrics and City of L.A. DoA. The L.A. CAPRA Community Action Board (CAB) motivated and guided the development of this project, identifying early on the need for a practical community-based stroke and physical activity program for older adults. The CAB worked closely with the study team to recruit laypersons and community representatives from each of the four targeted ethnic groups to serve on four ethnic-specific “mini-CABs” for the purposes of this project. The mini-CABs helped develop the intervention curriculum, iteratively reviewing drafts to ensure cultural specificity and sensitivity. Mini-CAB leaders also worked in close partnership with the UCLA-based team on all other project decisions including the recruitment and enrollment protocol, measurement selection, intervention design, and staff training.

### Worth the Walk intervention: conceptual basis and development

The Worth the Walk intervention is culturally-tailored and based in social cognitive theory [21, 22] and attribution theory [23, 24]. It incorporates elements such as verbal persuasion, goal setting, problem solving, and attribution retraining techniques that encourage participants to modify their expectations for aging (i.e., teach older adults not to attribute mutable stroke risk factors to aging) and change their own behavior to reduce stroke risk. In a previous National Institute on Aging-funded RCT of a behavioral intervention to increase walking—¡Caminemos! (R01 AG024460-05) [25, 26]—our team enrolled 572 older Latinos from 27 community senior centers and randomized them to receive either an attribution retraining intervention teaching not to attribute sedentary lifestyle to old age, or an active control group (series of lectures). We followed participants for 24 months; the intervention successfully increased walking levels (mean increase 6207 steps/day) more than the control group (*p* = 0.04). Though this efficacy study succeeded in meaningfully increasing walking levels, the intervention delivery was supported by National Institutes of Health (NIH) funding of research staff, limiting the intervention’s sustainability. With the current effectiveness trial, we have adopted many components of the Caminemos intervention, linking them with stroke and stroke risk factor education to create a new culturally-tailored intervention, and have integrated the new intervention directly into senior center programming without relying on NIH-funded staff for implementation (essentially making the efficacy-to-effectiveness transition). Our study builds on the expertise our team acquired in conducting the Caminemos trial, including recruitment, retention, screening and enrollment protocols, pedometer training, and group leader training (see below).

The Worth the Walk intervention consists of a group-based facilitated curriculum with 8 sessions covering topics such as racial/ethnic disparities in stroke and stroke outcomes, what stroke risk factors and warning signs are, blood pressure control, and seeing a healthcare provider regularly. Sessions are 1 h in duration and occur twice weekly for 4 consecutive weeks at a designated senior center. Information in the curriculum on stroke risk factor knowledge is based on materials developed by the American Heart Association/American Stroke Association (AHA/ASA). Special emphasis is placed on physical activity because of its substantial contribution to stroke risk on a population basis [16]. Walking is emphasized as an appealing physical activity based on its association with decreased morbidity and mortality among older adults [27] and the practical reality that walking is an accessible form of physical activity for most seniors.

Case manager group leaders provide verbal reinforcement of the concept that mutable risk factors for stroke such as sedentary lifestyle and high blood pressure should not be attributed to “old age,” and that all participants should expect to decrease their risk of stroke. At the end of alternating group sessions, group leaders encourage participants to set individualized verbal and written “promises” for improving their stroke risk factors – especially walking. Then, at the start of each subsequent group discussion session, participants are asked to report to the group: 1) the extent to which they met their personal promises; 2) whether there have been any difficulties in following through on their promises; 3) whether their beliefs about their ability to improve their stroke risk factors have changed since they started the program. Group leaders then provide positive encouragement and reinforce the message that *controlling* stroke risk factors should be an expected part of aging. Case manager group leaders also teach participants to identify the individual reasons why they might not always keep their stroke risk factor reduction promises, determine which reasons are mutable, and then “problem solve” [28] to identify solutions. For example, one reason for not reaching a walking goal could be that participants feel it is “too hot.” Though weather per se is not mutable, participants can come up with possible solutions to this problem, such as walking in the early morning, walking indoors at a shopping mall, etc. Problem-solving is generated from the group itself as much as possible; the role of the case manager group leaders is to help participants generate their own solutions. Knowledge, therefore, will be generated and conveyed within the group itself. It is also supplemented by comments from the case manager group leaders, who provide detailed instruction on how to improve stroke risk factors in the context of problem-solving exercises [29]. Finally, participants are provided with a diary (adapted from AHA/ASA materials) where they are encouraged to record their daily physical activity. Participants experience performance accomplishment (internal positive reinforcement of behavior) when they follow through on their “promises” and observe themselves meeting their goals in their personal diaries.

To guide the development and cultural tailoring of WTW and ultimately enhance its acceptability, relevance, and impact, we conducted 12 focus groups with a sample of 132 African American, Latino, Chinese, and Korean seniors in Los Angeles prior to the trial. Based on these findings, culturally-specific beliefs about stroke and its risk factors were incorporated into the intervention curriculum using Hwang’s Formative Method for Adapting Psychotherapy (FMAP) [30]. Operationalizing this framework allowed us to plan and document cultural adaptations to the curriculum in a systematic manner around the domains of cultural beliefs, curriculum/intervention orientation, participant-moderator relationship, and cultural issues of salience. Sessions 6 and 7 of the curriculum were specifically tailored for each of the four targeted ethnic groups and contain different subject matter based on the focus group data; for example, African American sessions 6 and 7 are entitled “Walking is Good for the Body (and Relieving Stress)” and “Walking is Good for the Soul,” respectively, while for Chinese Americans they are entitled “The ‘3 Highs’: High Blood Pressure, High Cholesterol, High Fat” and “Family Matters.” The 4 ethnic-specific mini-CABs iteratively modified the intervention to increase its likelihood of resonating with targeted end users and also advised on how its relevance and impact could be improved overall.

Prior to intervention implementation, all case managers completed an intensive 2-day training session led by the study team during which ethnic-specific versions of the curriculum and an overview of the study design were covered. Two case managers were trained per site. Certificates of completion were distributed at the conclusion of the training when case managers were able to successfully lead an observed mock session and demonstrate their ability to teach the content accurately.

### Setting

To maximize the sustainability of the intervention and achieve effectiveness research conditions, we integrated the WTW intervention into existing programming at 4 senior centers in Los Angeles. Los Angeles is an ideal place to conduct this study, not only because of its exceptional aging services network, but also because of its extraordinary ethnic diversity and leadership in the U.S. sociodemographic shift towards non-majority race being the norm. Los Angeles is home to large populations of three of the fastest growing demographic groups of older Americans: Latinos, Chinese Americans and Korean Americans, who together comprise over 50 % of the total population in Los Angeles County (ethnic-specific populations as a whole, not just those aged 65 years and older) [31].

### Participants – inclusion and exclusion criteria

We are enrolling an eventual total of 240 participants (60 participants from each of the 4 targeted ethnic groups). Eligibility criteria include: 1) age 60 years or older; 2) self-identifying as the racial/ethnic group for the intervention planned at that site (African American, Latino, Chinese, or Korean); 3) ability to communicate verbally in the appropriate language (English, Spanish, Mandarin, or Korean) in a group setting; 4) ability to sit in a chair and participate in a 1 h discussion session; 5) ability to walk with or without the use of assistive devices such as canes and walkers; 6) available to attend the baseline data collection session and subsequent weekly intervention sessions; 7) has been told by a health care provider that s/he has high blood pressure; 8) able/willing to provide the name of a physician who has seen the potential participant in the past 6 months and provide signed consent for our staff to contact this physician; 9) plans to continue to live in the region during the next 6 months; and 10) cognitive capacity to provide informed consent to participate. Potential participants whose physicians fax a reply card indicating medical contraindication are not eligible (see below under Study procedures, Recruitment section). Potential participants whose physicians fax a reply card indicating no contraindication or do not respond within one week are eligible.

### Study procedures

#### Recruitment, screening, enrollment, baseline

Recruitment takes place at the same 4 senior centers (one for each ethnic group) where in-house WTW case managers have been trained. Members of the research team make brief presentations describing the project during events attended by large numbers of seniors, such as the low-cost hot midday meals provided at all of the senior centers participating in this study. Recruitment flyers our team developed in partnership with our mini-CABs listing the study objectives, eligibility criteria, and staff contact information are also posted and distributed at senior center sites. Potentially eligible seniors are invited to approach/contact study staff individually to be formally screened on pre-determined days at the senior center.

During screening, study staff complete in face-to-face format a screener form listing the eligibility criteria with each prospective participant. Prospective participants are given a FITBIT® Zip pedometer and instructed on its proper use. A letter and reply form (referenced above) is then faxed to the physician indicated by each potentially eligible participant (along with the patient’s signature indicating consent for our staff to contact the physician) describing the intervention and asking him or her to contact the study team within one week stating whether his or her patient has any medical contraindication to participating, including unstable angina, uncompensated heart failure, uncontrolled cardiac arrhythmia, severe aortic stenosis, hypertrophic cardiomyopathy, cardiomyopathy from recent myocarditis, severe pulmonary hypertension, abdominal aortic aneurysm, recent systemic or pulmonary embolus, thrombophlebitis, and severe balance problems. A faxable reply form is provided so that the physician can simply check “yes” or “no.”

Approximately one week after screening has been completed, eligible participants are invited to attend a baseline data collection session at the senior center, during which trained project staff complete the informed consent process with each potential participant individually in a private setting. After participants have provided written documentation of informed consent, staff proceed to collect baseline data (see Table 1).

**Table 1:** Data collection measurements

Construct	Measurement	When measured			Source
Primary outcomes					
Walking level	Mean steps/day over 1 week	T0	T1	T2	Pedometer
Physical activity	International Physical Activity Questionnaire (IPAQ)	T0	T1	T2	Survey
Stroke and stroke risk factor knowledge	Stroke Action Survey (STAT)	T0	T1	T2	Survey
	Stroke risk factor knowledge				
Self-efficacy	General Self-Efficacy Scale	T0	T1	T2	Survey
	Chronic Disease Self-Efficacy Scale				
	Outcome Expectations for Exercise Scale (OEE)				
Secondary/exploratory outcomes					
Blood pressure	Universal data collection protocol	T0	T1	T2	Physical exam
BMI (kg/m2)	Universal data collection protocol	T0	T1	T2	Physical exam
Non-HDL cholesterol	Point-of-care CardioChek meter	T0		T2	Fingerprick
HDL cholesterol	Point-of-care CardioChek meter	T0		T2	Fingerprick
Triglycerides	Point-of-care CardioChek meter	T0		T2	Fingerprick
Glycosylated hemoglobin (HgA1c)	Dried blood spots	T0		T2	Fingerprick
c-reactive protein (CRP)	Dried blood spots	T0		T2	Fingerprick
Healthcare seeking	Visits with healthcare provider	T0		T2	Survey
Restricted bed days	Restricted activity	T0		T2	Survey
Social support/network	Interpersonal Support Evaluation List (ISEL)	T0	T1	T2	Survey
Health-related quality of life	Medical Outcomes Study SF-12	T0	T1	T2	Survey
Depressive symptoms	Patient Health Questionnaire (PHQ-9)	T0	T1	T2	Survey
Disability	ADL Summary Scale	T0	T1	T2	Survey
Risk perception and worry	SPRITE survey items	T0	T1	T2	Survey
Trust in physicians	Trust in Physicians Scale	T0	T1	T2	Survey
Trust in medical researchers	Trust in Medical Researchers	T0	T1	T2	Survey
Sleep	MOS-Sleep Scale	T0	T1	T2	Survey
Stress	Perceived Stress Scale-4 item	T0	T1	T2	Survey
Aging expectations	Expectations Regarding Aging (ERA-12)	T0	T1	T2	Survey
Other measures^a^					
Demographics/SES	REDCap database questions on demographics/highest completed education	T0			Survey
Acculturation^b^	Vancouver Index of Acculturation (VIA)	T0			Survey
Medical comorbidities	Katz/Charlson Comorbidity Index	T0			Survey
Neighborhood walkability	Neighborhood Environment Walkability Scale-Abridged (NEWS-A)	T0			Survey
Smoking	NHANES survey items	T0			Survey

*T0* baseline, *T1* 30 day follow-up, *T2* 90 day follow-up

^a^ These constructs, though not linked explicitly to specific aims, are considered to be critical covariates, likely to be associated with outcomes, which will be measured to evaluate the success of the randomization

^b^ Measured for non-African American participants only

#### Randomization & blinding

After baseline data have been collected and participants have dispersed, we utilize a computerized randomization procedure stratified by site and gender to assign participants to either the intervention or the control (wait-list) arm. The randomization procedure is programmed into Research Electronic Data Capture (REDCap), a secure web application for building and managing online surveys and databases [32]. A randomization allocation table was created using random permuted blocks with randomized block sizes 2, 4, 6 and is uploaded into REDCap. Smaller block sizes are used to distribute participants within either group as evenly as possible.

One designated research assistant is un-blinded for the duration of the study and works with senior center staff to schedule the WTW intervention sessions. Senior center staff call participants to inform them of their scheduled intervention session. While the case manager group leaders administer WTW, the same un-blinded research assistant attends sessions to take attendance and monitor fidelity to the curriculum using a standardized checklist of all the major teaching points of each intervention session (see Additional file 1: Appendix A). Other (blinded) project staff members maintain regular telephone contact with participants in both arms of the study to remind them to wear their pedometers but without explicitly prompting physical activity or walking. Project staff who are in the field collecting data are blinded to the participant assignment group for the entire study duration.

All participants in both the intervention and control groups receive the same frequency of contact from study staff (phone call reminders) and the same incentives (pedometers, $25 visa gift card at each data collection time point). We do not refer to the control group as a “wait-list” when speaking with participants and agency staff, but rather refer to that group by the month in which they will start the WTW program, for example, we will compare the “January” group with the “April” group.

### Study assignments

#### Intervention group

Participants assigned to the intervention group immediately attend the group-based interactive behavioral stroke risk reduction walking intervention led by an in-house case manager after baseline (T0). In addition to reminder phone calls prior to each intervention session, efforts to increase retention and sustain participation in the intervention include scheduling transportation for participants when applicable, as well as notifying participants that attendance will be taken at each session.

#### Wait-list control group

Participants assigned to the wait-list control group are invited to participate in WTW after follow-up data collection is completed 3 months from baseline (they do not repeat outcome measures at this time). As previously mentioned, control group participants are also given pedometers by blinded RAs, receive the same reminders to wear them, have follow-up data collected at the same time points, and have the same frequency of contact with study personnel as the intervention group throughout the study duration.

### Measurements

#### Data sources

Data sources include FITBIT® Zip pedometers [33], surveys, physical exams, and fingerpricks. The surveys, physical exams, and fingerpricks are administered with each individual at the senior centers by trained bilingual study staff. Surveys are interviewer-administered using REDCap [32], accessed using iPads with internet access. Survey instruments were translated into the native languages of our targeted ethnic groups (Spanish, Mandarin and Korean) a priori through a professional translation company; the accuracy of translations was verified by bilingual study staff as well as by seniors with whom the surveys were piloted prior to trial implementation. Pedometer data are downloaded using the iPads and stored/accessed through the secure FITBIT® website at each data collection time point. All study outcomes, collection time points, and data sources are listed in Table 1.

#### Primary outcomes

The primary outcome measure is change in walking level from baseline (T0) to 1 and 3 months (T1 and T2) as measured by the FITBIT® Zip pedometers. Physical activity level is supplementarily assessed using the International Physical Activity Questionnaire [34] included in the survey at all three time points. Stroke risk factor knowledge is assessed via survey using the Stroke Action Survey and questions developed by Pancioli et al. [35, 36]. We measure self-efficacy for reducing stroke risk and increasing physical activity. Self-efficacy is best captured by measuring both self-efficacy expectations and outcome expectations [21]. As such, we measure self-efficacy expectations with the General Self-Efficacy Scale [37] and a modified version of the Chronic Disease Self-Efficacy Scale [38] that focuses on self-efficacy for stroke risk reduction; outcome expectations for being physically active is assessed using the Outcome Expectations for Exercise Scale [39].

#### Secondary/exploratory outcomes

To explore the relationship between the intervention and biological markers of health, we collect height and weight in light clothing without shoes at all three data collection time points in strict accordance with standardized procedures we have used previously. A random 5 % of participants will have their weight and height measured by two different research assistants so that inter-rater reliability can be calculated. To assess our main secondary outcome measure—systolic blood pressure—trained staff implement a standard, seated blood pressure protocol based on current Joint National Committee on Prevention, Detection, Evaluation, and Treatment of High Blood Pressure (JNC 7) guidelines, but allowing a 5-min rest between each measure [40]. Point-of-care CardioChek meters are used to measure cholesterol (using the Lipid Panel strip), which require only a small capillary blood sample (from a fingerprick). We use dried blood spots to obtain assays for HgA1c and CRP, also collected by fingerpricks. So that we can explore whether the intervention influenced healthcare seeking to control stroke risk factors, we ask participants to report on visits to healthcare providers in the previous three months at baseline and 3 months [41].

#### Other exploratory outcomes and covariates

Other measures being collected are listed in Table 1. These include exploratory outcomes that might be influenced by the intervention such as health-related quality of life [42] and stress [43]. In addition, we will measure constructs such as acculturation level [44] and neighborhood walkability [45] that, while not linked explicitly to specific aims, are considered to be critical covariates likely to be associated with outcomes and can also be used to evaluate the success of the randomization.

### Sample size and power

As described above, our primary outcome of interest is change in mean steps/day, while our main secondary outcome measure is change in systolic blood pressure. Based on our capacity, we plan to enroll 240 subjects from 4 senior centers. From our previous senior center research we estimate that as many as 15 % of subjects will not complete follow-up. Data from our previous senior center studies of older Latinos and African Americans showed mean steps/day 2713 (std. dev. 2190) and mean SBP 141 mm Hg (std. dev. 20 mm Hg); intra-class correlation within senior centers was at 0.0085. Since we propose a repeated measures design with 3 repeated measurements [46], the underlying statistical power of the study comes from two dimensions of observations: the number of unique subjects and the number of repeated measurements within a subject. Based on sample size/power analytic methods for repeated measures analysis [47, 48], using a 2-sided test with a type I error of 0.05, and a type II error of 0.2 (power 80 %), and assuming an average 2.7 data points per subject and an auto-correlation at 0.2 level, after adjusting for clustering, the effective sample size will be 96 subjects in each arm, which will enable us to detect effect sizes as small as 581 steps/day (far below a clinically meaningful increase of 5000 steps/day [49]) and 5.3 mm Hg. A decrease of 5 mm Hg substantially decreases stroke risk [50] and is comparable to effect sizes observed in walking interventions of similar intensity [51].

### Statistical analyses

#### Overview of analytic plan

We will use standard analytic methods for randomized controlled trials [52]. After comparing baseline characteristics of intervention and control group participants, and accounting for missing data, outcomes will be compared between the two groups. Though we have made unidirectional hypotheses, to allow for the chance that participants enrolled to the control group could have better outcomes than those in the intervention arm, we will use 2-tailed tests of significance for all analyses.

#### Multiple comparisons [53]

We have a priori selected the outcome of change in steps/day to be the primary endpoint of interest. Analyses of all other outcomes will be adjusted for multiple comparisons [54].

#### Intention to treat

All analyses will be conducted using intention to treat, in which any subject randomized to the intervention arm remains in that arm regardless of whether or not he or she received the intervention, and likewise for the control arm. We will measure level of participation for those randomized to the intervention arm, and will conduct a sensitivity analysis that assesses the stability of the study’s conclusions when an intention to treat analysis versus an analysis that takes into account level of participation in the intervention.

#### Comparing baseline characteristics of intervention and control arm subjects

Using t-tests for continuous variables and chi-square tests for categorical variables, we will compare the two groups on baseline characteristics to measure the success of the randomization: 1) sociodemographic characteristics; 2) mean steps/day over 1 week in the window post screening and distribution of pedometers, to the T0 baseline data collection one week later; 3) systolic blood pressure, body mass index, non-HDL cholesterol, HgA1c; 4) stroke knowledge, self-efficacy. Since steps/day is a continuous variable, at each time-point (30 days/T1 and 90 days/T2) we will perform a simple descriptive cross-sectional analysis of mean change in scores between baseline and follow-up in each treatment arm. In our primary analyses, we will test the statistical significance of the unadjusted difference in change-scores between the groups using t-tests for symmetrically distributed data and analogous nonparametric tests such as the Wilcoxon sign rank tests for data that are skewed. Imbalance between the treatment arms of characteristics described above would inflate the standard error, making the unadjusted analyses appropriately conservative; therefore, the unadjusted analyses will serve as the primary results of the trial. If, however, we did identify chance differences between the treatment arms in the distribution of the baseline level of the outcome, then we will also conduct secondary analyses in which we will use multivariate modeling, such as analysis of covariance, to adjust for these factors. The adjusted means of the change-scores from multivariate models will be compared and tested between the two arms, and will also be compared with the unadjusted means to assess the influence of these factors on endpoints. As sensitivity analyses, we will also examine the difference between groups in the absolute steps/day (instead of the change-score), adjusting for baseline. These sensitivity analyses will allow us to compare outcomes between groups without assuming linearity of effect across different baseline levels of physical activity. Using multivariate modeling, we will also test interactions between treatment arm and selected effect modifiers (e.g., age, level of acculturation, etc.) to identify sub-groups of participants who improve more than other groups. Since this trial is not designed with power to examine these subgroups, these analyses will be exploratory and hypothesis-generating in nature. Finally, we will assess changes in steps/day between baseline and the end of the intervention period, and differences between the control and intervention groups in steps/day trajectories over time. These analyses will consist of graphical analysis and parametric statistical testing using repeated measures mixed effects models [55, 56]. To determine the extent to which increases in stroke knowledge and self-efficacy mediate improvements in steps/day, the variation (R-squared) in increase in steps/day is explained by variation in increase in the stroke knowledge and self-efficacy constructs will be calculated [57, 58]. Analysis of the effect of the intervention on all the secondary and exploratory outcomes will follow the same analysis.

### Process evaluation

We will examine the variation in effectiveness of the intervention among the 4 sites and will conduct a formal process evaluation to: 1) determine general and site-specific factors associated with greater effectiveness; 2) assess the feasibility, acceptability, and sustainability of the intervention; 3) measure the costs needed to implement and maintain the intervention. We will meet separately with key stakeholders at each site including seniors (RCT participants), case manager group leaders, senior center directors, other senior center staff involved in implementing the program (for example the staff members who set the schedules at the senior centers), and 2–3 healthcare providers involved in the enrollment screening process for participants at each site. In semi-structured interviews guided by previous successful implementation analyses [59, 60] we will ask questions such as: What was your role in the intervention and what outcomes did you seek to achieve? How was the intervention received by you and others in your site and did this change over time? What are the potential barriers and facilitators to implementing the intervention outside of the research study? What problems were associated with delivering the intervention and how might they translate (or not) into “real-world” implementation beyond the study trial (NIH grant) period? How did this program affect workload, burden and space? What potential modifications to the intervention could be made to maximize implementation? How (if at all) did this program change your relationship with the healthcare/senior center community? Transcripts will be read by 2 investigators who will use previously-described content analysis methods [60, 61] to identify behaviors, attitudes, personal characteristics, contexts, processes, and policies the informants believed to be associated with the implementation and sustainability of the intervention. Start-up costs needed to implement the intervention (such as items required for group leader training) will be tracked and recorded.

## Discussion

Results from this trial will provide important insight into the design and effectiveness of sustainable community-based interventions aiming to reduce stroke risk and mitigate disparities among hypertensive ethnic minority seniors. We have combined two different theories from motivational psychology with an extensive body of stroke education using materials from the AHA/ASA to develop a culturally-tailored intervention that has been directly integrated into the aging services network in Los Angeles. The demographic composition of Los Angeles is representative of projected trends for the nation; therefore, culturally-tailored projects that succeed in Los Angeles are natural models to take to scale nationally.

We recognize that a major disadvantage to using a wait-list control rather than an active simultaneous control is that participants are not blinded to whether or not they are receiving the intervention and thus may have different expectations of improvement during the data collection period. In other words, participants in the intervention arm might improve just because they know they are in the intervention arm rather than from the content of the intervention (and conversely those on the wait list might show little improvement because they know they are not “supposed” to improve while on the wait list). In addition, there could be disproportionate follow-up between study arms as wait-list participants move away or lose interest that could bias the study findings. Alternatively, participants randomized to the wait list control arm could seek their own plan to prevent stroke risk outside of the intervention, contaminating the control arm.

Nevertheless, given the strong level of enthusiasm by senior center leaders and clients (seniors) for this type of intervention, it is not feasible to have a blinded control arm within a single senior center; this would be perceived as unfair and generating ill-will among the senior center attendees. To minimize the effect of non-blinding on the study design, we ensure that all participants receive the same frequency of contact from study staff in terms of phone call reminders and incentives including pedometers.

Though multifaceted (including both formal stroke education and behavioral change strategies), this intervention is intentionally designed to be low-cost, sustainable and scalable. We chose to use in-house AAA-funded case managers rather than professional healthcare providers because a major goal of this project is to evaluate a practical and sustainable intervention that can be scaled up across the national aging services network; thus, even a modest stroke risk factor reduction in physical activity or blood pressure could have a tremendous population impact on preventing strokes and decreasing stroke disparities. Upon completion of this trial, we will have trained a cadre of senior-center based case managers in a new skill set of health disparities intervention implementation that can be used not only for stroke risk factor reduction interventions but also other evidence-based health promotion programs.

## Additional file

Additional file 1:
**Appendix A.** Worth the Walk: African American Curriculum Fidelity Measurement Tool.

## Abbreviations

RCT: Randomized controlled trial
L.A.: Los Angeles
DoA: Department of aging
AAA: Area agency on aging
ACL: Administration for community living
WTW: Worth the Walk
HgA1c: Glycosylated hemoglobin
CRP: c-reactive protein
L.A. CAPRA: Los Angeles community academic partnership for research in aging
CAB: Community action board
NIH: National institutes of health
AHA/ASA: American heart association/American stroke association
FMAP: Formative method for adapting psychotherapy
REDCap: Research electronic data capture
