# Supplementary material for: Study protocol of “Worth the Walk”: a randomized controlled trial of a stroke risk reduction walking intervention among racial/ethnic minority older adults with hypertension in community senior centers
Source: BMC Neurol. 2015 Jun 15;15:91. doi: 10.1186/s12883-015-0346-9 (PMC4465734; doi:10.1186/s12883-015-0346-9)
Supplement: Additional file 1: — Appendix A. Worth the Walk: African American Curriculum Fidelity Measurement Tool. [file 12883_2015_346_MOESM1_ESM.docx]

**Additional file 1: Appendix A**

**Worth the Walk: African American Curriculum Fidelity Measurement Tool**

**Session 1 Why Walking's Worth it**

Did the Case Manager do the following?

- Introduce themselves to the group and participants to each other.
- Introduce the concept of stroke.
- Introduce the idea that being physically active and controlling stroke risk factors should be an expected part of normal aging and should continue at any age.
- Focus on physical inactivity as a risk factor and identify causes of being less physically active.
- Differentiate between causes of physical inactivity that are modifiable and causes that are not (like age).
- Teach that aging itself does not cause stroke or decreased physical activity.
- Discuss Women-specific teaching point #1
- **Make individual promises to improve stroke risk factors through increasing walking and physical activity.**
- **Distribute and explain stroke fact sheet**

**Session 2 What’s Worth Looking Out For**

Did the Case Manager do the following?

- Introduce stroke warning signs.
- Reinforce the idea that preventing stroke and being physically active should be an expected part of normal aging and should continue at any age.
- Reinforce physical activity as a modifiable risk factor for stroke and the difference between modifiable and non-modifiable contributors to being less physically active.
- Identify common changes with aging and teach that modifications can make activity once again possible.
- Discuss Women-specific teaching point #2
- **Introduce/distribute diary and reminder to keep up with promises.**

**Session 3 Worth the Talk: Me and My Doc**

Did the Case Manager do the following?

- Review promises and problem-solve on barriers to completion.
- Reinforce the idea that knowing stroke symptoms and being physically active are modifiable risk factors for stroke and should be an expected part of normal aging.
- Identify common challenges with communicating with your doctor.
- Problem solve solutions or ways to manage these challenges.
- **Make new promises.**

**Session 4 Taking Control, One Step at a Time**

Did the Case Manager do the following?

- Introduce blood pressure control.
- Reinforce the idea that preventing stroke and being physically active should be an expected part of normal aging and should continue at any age.
- Reinforce the idea that difficulty walking and controlling stroke risk should not be attributed to old age.
- Teach about importance of incremental goal setting.
- Problem solve on how to avoid feeling overwhelmed when trying to manage stroke risk.
- **Reminder to keep up with promises.**

**Session 5 It’s Never Too Late to Make Walking Fun and Worth It**

Did the Case Manager do the following?

- Review promises and problem-solve on barriers to completion.
- Reflect in a meaningful way on whether expectations and beliefs about aging have changed.
- Teach that being unable to learn a new habit is not caused by aging.
- Present Women-specific teaching point #3
- Problem-solve on how to establish an exercise or walking plan as a new habit.
- **Make new promises.**

**Session 6 Walking is good for the Body (and Relieving Stress)**

Did the Case Manager do the following?

- Review stroke warning signs.
- Reflect in a meaningful way on whether expectations around aging and habit formation have changed.
- Teach about chronic emotional stress and stroke risk.
- Problem-solve on how walking can be used to reduce emotional stress and stroke risk.
- Teach that we have control over how we choose to cope with stress, and that walking is an excellent choice.
- **Reminder to keep up with promises.**

**Session 7 Walking is good for the Soul**

Did the Case Manager do the following?

- Review promises and problem-solve on barriers to completion.
- Reflect in a meaningful way on whether expectations and beliefs about aging have changed.
- Teach that pairing walking with a favorite routine activity and walking with others can help make exercise a new habit.
- Problem-solve on ways to incorporate walking into your regular routine and also on ways to involve family and friends.
- Discuss Women-specific teaching point #4
- **Make new promises.**

**Session 8 My Time to Shine**

Did the Case Manager do the following?

- Review progress with promises and problem-solve on barriers to completion.
- Reflect in a meaningful way on whether expectations and beliefs about aging have changed.
- Reinforce the idea that being physically active should be an expected part of normal aging and should continue at any age.
- Reinforce the idea that difficulty walking should not be attributed to old age.
- Identify good things about getting older.
- Problem solve on how to maintain an exercise or walking plan.
- **Make new promises for the next month.**
